# Assessing the Utility of Prediction Scores PAINT, ISARIC4C, CHIS, and COVID-GRAM at Admission and Seven Days after Symptom Onset for COVID-19 Mortality

**DOI:** 10.3390/jpm14090966

**Published:** 2024-09-11

**Authors:** Alina Doina Tanase, Oktrian FNU, Dan-Mihai Cristescu, Paula Irina Barata, Dana David, Emanuela-Lidia Petrescu, Daliana-Emanuela Bojoga, Teodora Hoinoiu, Alexandru Blidisel

**Affiliations:** 1 Department of Professional Legislation in Dental Medicine, Faculty of Dental Medicine, “Victor Babes” University of Medicine and Pharmacy, Eftimie Murgu Square 2, 300041 Timisoara, Romania; tanase.alina@umft.ro; 2 Doctoral School, “Victor Babes” University of Medicine and Pharmacy, Eftimie Murgu Square 2, 300041 Timisoara, Romania; dancristescu93@gmail.com; 3 Faculty of Medicine, University of Indonesia, Jakarta 10430, Indonesia; oktrian@alumni.ui.ac.id; 4 Research Centre of Timisoara Institute of Cardiovascular Diseases, “Victor Babes” University of Medicine and Pharmacy, 300041 Timisoara, Romania; 5 Center for Research and Innovation in Precision Medicine of Respiratory Diseases, “Victor Babes” University of Medicine and Pharmacy, Eftimie Murgu Square 2, 300041 Timisoara, Romania; 6 Department of Physiology, Faculty of Medicine, “Vasile Goldis” Western University of Arad, 310025 Arad, Romania; 7 Discipline of Biochemistry, “Victor Babes” University of Medicine and Pharmacy, Eftimie Murgu Square 2, 300041 Timisoara, Romania; david.dana@umft.ro; 8 Department of Prostheses Technology and Dental Materials, Faculty of Dental Medicine, “Victor Babes” University of Medicine and Pharmacy, Eftimie Murgu Square 2, 300041 Timisoara, Romania; petrescu.emanuela@umft.ro; 9 Research Centre in Dental Medicine Using Conventional and Alternative Technologies, Faculty of Dental Medicine, “Victor Babes” University of Medicine and Pharmacy, Eftimie Murgu Square 2, 300041 Timisoara, Romania; 10 Department of Oral Rehabilitation and Emergencies in Dental Medicine, Faculty of Dental Medicine, “Victor Babes” University of Medicine and Pharmacy, Eftimie Murgu Square 2, 300041 Timisoara, Romania; mocuta.daliana@umft.ro; 11 Interdisciplinary Research Canter for Dental Medical Research, Lasers and Innovative Technologies Faculty of Dental Medicine, “Victor Babes” University of Medicine and Pharmacy, Eftimie Murgu Square 2, 300041 Timisoara, Romania; 12 Department of Clinical Practical Skills, “Victor Babes” University of Medicine and Pharmacy, Eftimie Murgu Square 2, 300041 Timisoara, Romania; tstoichitoiu@umft.ro; 13 Clinic of Surgical Semiotics and Thoracic Surgery—1, Department IX—Surgery—1, “Victor Babes” University of Medicine and Pharmacy, Eftimie Murgu Square 2, 300041 Timisoara, Romania; blidy@umft.ro; 14 Center for Hepato-Biliary-Pancreatic Surgery (CHBP), “Victor Babes” University of Medicine and Pharmacy, Eftimie Murgu Square 2, 300041 Timisoara, Romania

**Keywords:** COVID-19, SARS-CoV-2, prediction, mortality

## Abstract

The COVID-19 pandemic underscores the need for accurate prognostic tools to predict patient outcomes. This study evaluates the effectiveness of four prominent COVID-19 prediction scores—PAINT, ISARIC4C, CHIS, and COVID-GRAM—at two critical time points: at admission and seven days post-symptom onset, to assess their utility in predicting mortality among hospitalized patients. Conducted at the Clinical Emergency Hospital Pius Brînzeu in Timișoara, this retrospective analysis included adult patients hospitalized with confirmed SARS-CoV-2 infection. Eligible patients had complete data for the scores at both time points. Statistical analysis involved ROC curves and logistic regression to assess the scores’ predictive accuracy for mortality. The study included 215 patients, split into 139 survivors and 76 non-survivors. At admission, the PAINT, ISARIC4C, CHIS, and COVID-GRAM scores significantly differentiated between the survival outcomes (*p* < 0.0001). The best cutoff values at admission were 6.26 for PAINT, 7.95 for ISARIC4C, 5.58 for CHIS, and 0.63 for COVID-GRAM, corresponding to sensitivities of 85.47%, 80.56%, 88.89%, and 83.33% and specificities of 77.34%, 82.12%, 75.01%, and 78.45%, respectively. By day seven, the cutoff values increased, indicating deteriorating conditions in patients who eventually succumbed to the virus. The hazard ratios at admission for exceeding these cutoffs were significant: PAINT (HR = 3.45), ISARIC4C (HR = 2.89), CHIS (HR = 4.02), and COVID-GRAM (HR = 3.15), highlighting the scores’ abilities to predict severe outcomes. One week post symptom onset, these scores’ predictive values and corresponding hazard ratios increased, further validating their prognostic significance over time. The evaluated COVID-19 prediction scores robustly predict mortality at admission and become more predictive by the seventh day of symptom onset. These findings support the use of these scores in clinical settings to facilitate early identification and intervention for high-risk patients, potentially improving patient outcomes during the ongoing global health crisis.

## 1. Introduction

The coronavirus (COVID-19) pandemic, which erupted in late 2019, has escalated into a relentless global crisis. As of 2024, it has resulted in over 800 million confirmed cases and claimed more than 7 million lives worldwide, severely stretching the capacities of health systems across continents [1,2,3]. The economic impact has been staggering, with global economic losses estimated at approximately USD 10 trillion due to healthcare expenditures, disruptions in trade, tourism, and widespread job losses [4,5,6]. As the virus continued to mutate and spread, healthcare professionals have been compelled to rapidly identify patients at elevated risk of severe outcomes, including mortality [7,8]. This critical need has propelled the advancement of various clinical prediction tools, which have become essential in stratifying patients based on their likelihood of severe disease progression, thereby enabling more targeted and timely medical interventions.

The use of prediction scores in clinical settings has become a cornerstone for managing COVID-19 patients [9]. These tools integrate various patient data points to forecast the risk of severe disease, yet their effectiveness can vary based on demographic changes, virus mutations, and differing healthcare settings [10]. This variability highlights the need for ongoing research to validate and refine these tools under current pandemic conditions.

Other widely recognized tools and biomarkers have also played significant roles in the clinical management of COVID-19. These include the Sequential Organ Failure Assessment (SOFA) score [11], which evaluates organ dysfunction or failure. The NLR score (Neutrophil to Lymphocyte Ratio) [12], for instance, is a simple yet effective marker of systemic inflammation that has been linked to poor outcomes in COVID-19 patients. Similarly, the PLR (Platelet to Lymphocyte Ratio) provides insights into the balance of prothrombotic and inflammatory states, which are critical in severe COVID-19 infections [13]. Another important score is the CURB-65 (confusion, uremia, respiratory rate, BP, age ≥ 65 years) [14], which integrates confusion, respiratory rate, blood pressure, and age over 65, to help assess pneumonia severity and the inflammatory burden it imposes. The dynamic nature of the pandemic necessitates a multifaceted approach in utilizing these markers and scores, continually adapting to new evidence and emerging strains of the virus.

Moreover, understanding the temporal dynamics of COVID-19, particularly the changes in patient condition after the onset of symptoms, is critical. The first week following symptom onset is particularly pivotal, as it often determines the disease’s progression towards either recovery or severe complications [15,16]. Therefore, assessing the utility of predictive scores not only at admission but also during this critical period can provide valuable insights into their practical utility and reliability.

Given this backdrop, our study aims to evaluate and compare the effectiveness of several well-regarded COVID-19 prediction scores—namely PAINT, ISARIC4C, CHIS, and COVID-GRAM—at two key time points: at admission and seven days post symptom onset. Through this analysis, we intend to ascertain their predictive accuracy for mortality among hospitalized COVID-19 patients. This research could significantly impact clinical decision-making processes, contributing to improved patient outcomes during this ongoing global health crisis.

## 2. Materials and Methods

### 2.1. Legal and Ethical Considerations

The study was conducted according to the guidelines of the Declaration of Helsinki, and approved by the Local Ethics Committee of Timis County Emergency Clinical Hospital Pius Brinzeu OF Pius Brinzeu according to article 167 provisions of Law no. 95/2006, art. 28, chapter VIII of order 904/2006 and EU GCP Directives 2005/28/EC, International Conference of Harmonisation of Technical Requirements for Registration of Pharmaceuticals for Human Use (ICH) (protocol code 05 and date of approval is 28 February 2022). These guidelines stipulate rigorous standards for the ethical conduct of clinical research, emphasizing the protection of human subjects, the necessity of informed consent, and the requirement for scientific validity.

In compliance with the General Data Protection Regulation (GDPR) and other relevant national data protection laws, our study implemented strict measures to safeguard personal data. Patient information was anonymized prior to analysis to ensure confidentiality and to mitigate legal risks related to privacy breaches. Anonymization was performed by removing all direct and indirect identifiers that could link the data to an individual, thereby ensuring that data could not be re-identified according to legal standards.

The study protocols were also subject to legal review to ensure compliance with all applicable laws and regulations concerning clinical research. These included reviews related to the management of health data, ethical considerations specific to medical research, and compliance with international research standards. Regular audits were scheduled to monitor compliance, and any deviations from the approved protocols were to be reported immediately to the IRB.

The study upheld patient rights through transparent data handling procedures and strict adherence to ethical guidelines that prioritize patient welfare. Patients’ rights to privacy and data protection were treated as paramount throughout the research process. Additionally, the study’s findings are to be disseminated in a manner that respects the confidentiality of the data and the anonymity of the participants, ensuring that results are reported responsibly and ethically to contribute to broader medical knowledge without compromising individual privacy.

### 2.2. Inclusion and Exclusion Criteria

The study included adult patients aged 18 years and older who were hospitalized with a laboratory-confirmed SARS-CoV-2 infection, verified by RT-PCR testing. Eligible participants were required to have complete data available for the calculation of the PAINT, ISARIC4C, CHIS, and COVID-GRAM scores both at admission and seven days post symptom onset. Only patients with diabetes were selected to represent the co-morbid group. Exclusion criteria included patients who were transferred from other hospitals without initial scoring data, those who died or were discharged before the seven-day evaluation, and patients with incomplete records missing essential clinical parameters or outcomes. Cases where participants did not previously sign the consent for their medical records to be included in future medical studies were also excluded to respect patient consent and privacy.

### 2.3. Study Variables

For this study, we selected and collected specific variables at admission and again seven days after symptom onset to evaluate the predictive accuracy of the PAINT, ISARIC4C, CHIS, and COVID-GRAM scores for assessing mortality among hospitalized COVID-19 patients. The variables included demographic data such as age and gender; key clinical parameters like respiratory rate (RR), peripheral oxygen saturation (SpO_2_) of room air, and the Glasgow Coma Scale (GCS); and a detailed record of comorbidities that could influence COVID-19 progression. Additionally, essential laboratory values required for calculating the scores were gathered. These included blood urea nitrogen and C-reactive protein (CRP) levels, along with specific biomarkers relevant to each score. For instance, the PAINT score required data on IgM levels and NK cell counts (CD16+/CD56+), while the ISARIC4C score used variables such as blood urea nitrogen levels and CRP.

### 2.4. Definitions

The PAINT score is a predictive model specifically designed to estimate the risk of COVID-19 patients progressing from mild or moderate to severe disease. This score incorporates several clinical parameters: pulmonary disease, age, IgM levels, CD16+/CD56+ natural killer (NK) cells, and aspartate aminotransferase (AST). Each factor is assigned a specific weight based on its impact on disease severity. Pulmonary disease contributes 2.4174 points to the score, being over the age of 75 adds 1.3594 points, IgM levels below 0.84 contribute 1.8399 points, NK cell counts below 116.5 add 1.2246 points, and AST levels above 25 contribute 1.5182 points. These weighted contributions are summed to provide a total score, which can then be used to stratify patients according to their risk of developing severe complications, aiding clinicians in making informed decisions about the management and prioritization of treatment resources [17].

The ISARIC4C Mortality Score, developed to assess the risk of mortality in COVID-19 patients, is a comprehensive tool that utilizes a variety of clinical parameters collected at hospital admission. For calculation, this score considers several key variables: age, gender, number of comorbidities, respiratory rate (RR), peripheral oxygen saturation (SpO_2_) of room air, and the Glasgow Coma Scale (GCS). Additionally, the blood urea nitrogen level and C-reactive protein (CRP) measured at admission were included. These factors are quantitatively analyzed to produce a score that ranges from 0 to ≥15, categorizing patients into four distinct risk groups: low (0–3), intermediate (4–8), high (9–14), and very high-risk (≥15). This stratification helps healthcare providers identify patients at varying degrees of risk for severe outcomes, enabling prioritized and potentially life-saving interventions [18].

The COVID-19-associated Hyperinflammatory Syndrome (cHIS) score was developed from a comprehensive literature review comparing COVID-19 with other hyperinflammatory syndromes like secondary hemophagocytic lymphohistiocytosis. Based on identified similarities, cHIS integrates six core categories—fever, macrophage activation, hematological dysfunction, hepatic inflammation, coagulopathy, and elevated cytokine levels—each with specific laboratory biomarker thresholds indicative of severe disease. This additive six-point scale provides a structured method to assess the severity of hyperinflammation in COVID-19 patients, aiding in the timely management of critical cases [19].

The COVID-GRAM score is a predictive tool quantifying the risk of severe outcomes in hospitalized COVID-19 patients. It includes clinical parameters such as age, signs of dyspnea, comorbidities, and key laboratory findings like lymphocyte count and CRP levels, collected at hospital admission. The score ranges from 0 to 1, stratifying patients into three risk categories: low risk (0–0.41), medium risk (0.41–0.78), and high risk (0.78–1.00). This scoring system aids clinicians in assessing the potential severity of illness and tailoring appropriate management strategies for each patient [20].

### 2.5. Statistical Analysis

Data handling and statistical evaluations were conducted using SPSS Statistics version 25.0. Continuous data were represented as mean values ± standard deviation (SD), and categorical data were expressed in terms of frequencies and percentages. For the analysis of continuous variables among different groups, the Mann–Whitney U test was utilized due to the non-normal distribution of the clinical score data, while the Chi-square test was employed for categorical variables. Receiver operating characteristic (ROC) curves were generated to assess the predictive accuracy of the clinical scores, with the calculation of areas under the curve (AUC) and the determination of sensitivity and specificity values. Multiple logistic regression was applied to ascertain the odds ratios for severe COVID-19 outcomes based on the scores at admission and day five, with a *p*-value of less than 0.05 indicating statistical significance. The regression model was adjusted for potential confounders identified in preliminary analyses, including age, comorbidities, and baseline clinical severity.

We evaluated each of the clinical scores—PAINT, ISARIC4C, CHIS, and COVID-GRAM—separately in individual logistic regression models to avoid collinearity issues arising from overlapping variables across scores. Additionally, the coefficient of determination (adjusted R^2^) for each model was calculated to quantify how much of the variability in the dependent variable (severe COVID-19 outcomes) each model explains, thus providing a clear understanding of the predictive power and robustness of each score independently.

## 3. Results

In the current study, the comparison was set between survivors (*n* = 139) and the mortality group *(n* = 76). While age differences were not statistically significant, the mean ages were 57.23 years (SD = 14.56) for survivors and 60.89 years (SD = 10.42) for the mortality group (*p* = 0.0542), indicating a trend where those in the mortality group were slightly older. The BMI showed a significant difference, with survivors having a mean BMI of 27.68 (SD = 4.89) compared to 30.12 (SD = 6.03) in the mortality group, which was statistically significant (*p* = 0.0010).

Clinically, the differences were stark in outcomes that related to the severity of COVID-19. ICU admissions were significantly higher in the mortality group with 41 (53.95%) compared to only 5 (3.60%) in survivors (*p* < 0.0001). Similarly, the need for supplemental oxygen was dramatically higher in the mortality group, with 64 (84.21%) requiring it versus 18 (12.95%) among survivors (*p* < 0.0001). Mechanical ventilation was required for 39 (51.32%) patients in the mortality group compared to just 4 (2.88%) in the survivor group (*p* < 0.0001), as presented in Table 1.

Oxygen saturation levels were notably higher in the survivors’ group (94.32% ± 1.76) compared to the mortality group (85.47% ± 3.29), indicating severe respiratory compromise in the latter. The white blood cell count was markedly elevated in the mortality group (11.28 ± 4.26 × 10^9^/L) relative to the survivors (6.45 ± 1.34 × 10^9^/L), suggesting a more intense inflammatory response or infection. Lymphocyte counts were lower in the mortality group (0.82 ± 0.37 × 10^9^/L) compared to survivors (1.45 ± 0.48 × 10^9^/L), reflecting a possible lymphopenia associated with severe COVID-19 cases. Inflammatory markers and other laboratory parameters further underscored the severity in the mortality group; notably, ferritin (742.89 ± 330.45 ng/mL vs. 250.45 ± 110.78 ng/mL) and C-reactive protein levels (156.34 ± 70.56 mg/L vs. 20.78 ± 10.44 mg/L) were significantly higher, indicating heightened inflammatory states. PAINT scores were 7.84 ± 2.56 in the mortality group versus 3.25 ± 1.11 in survivors, ISARIC4C scores were 11.45 ± 3.67 versus 4.22 ± 1.34, CHIS scores were 8.34 ± 3.12 versus 2.56 ± 1.22, and COVID-GRAM scores were 0.89 ± 0.23 versus 0.34 ± 0.12 (*p*-value < 0.0001), as presented in Table 2.

One week post symptom onset, the analysis of clinical prediction scores provided a stark contrast between the survivors and the mortality group. The PAINT score, which incorporates factors like pulmonary disease, age, IgM levels, NK cell counts, and AST levels, was markedly higher in the mortality group (9.34 ± 3.45) compared to the survivors (2.87 ± 1.22), with a *p*-value of <0.001. This significant difference highlights its potential as a reliable indicator of disease progression. Similarly, the ISARIC4C score, which includes metrics such as age, comorbidities, and various clinical signs at admission, was also significantly elevated in the mortality group (14.56 ± 4.98) versus the survivors (3.65 ± 2.11), reflecting its effectiveness in mortality risk stratification.

The CHIS score, designed to evaluate hyperinflammatory states, stood at 12.67 ± 5.34 in the mortality group, much higher than the 1.98 ± 1.08 observed in the survivors. This score’s elevation is consistent with the severe inflammatory responses often seen in critical COVID-19 cases. Lastly, the COVID-GRAM score, which predicts the risk of severe outcomes based on clinical and laboratory parameters, was nearly double in the mortality group (0.98 ± 0.32) compared to the survivors (0.45 ± 0.23), as seen in Table 3.

**Figure 1:**
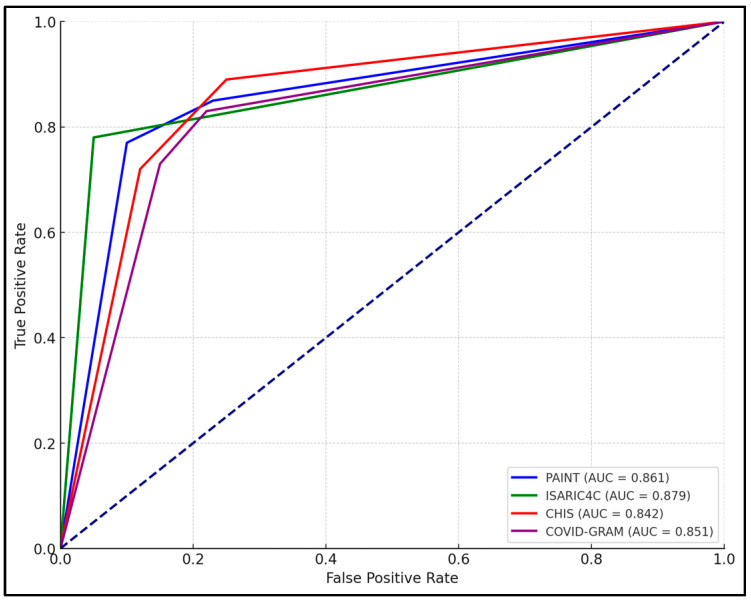
AUROC analysis of clinical prediction scores for COVID-19 mortality at initial measurement.

At baseline, the PAINT score demonstrated a robust ability to predict severe outcomes with a cutoff value of 6.26, achieving a sensitivity of 85.47% and specificity of 77.34%, with an AUC of 0.861 (*p* < 0.0001). This indicates a strong predictive power early in the disease course. The ISARIC4C score, with a cutoff of 7.95, also showed high effectiveness, marked by a sensitivity of 80.56% and a slightly higher specificity of 82.12%, reflected in an AUC of 0.879 (*p* < 0.0001). The CHIS and COVID-GRAM scores at baseline similarly demonstrated significant predictive value, with CHIS showing the highest sensitivity (88.89%) among the scores, albeit with a lower specificity of 75.01% (Figure 1).

**Figure 2:**
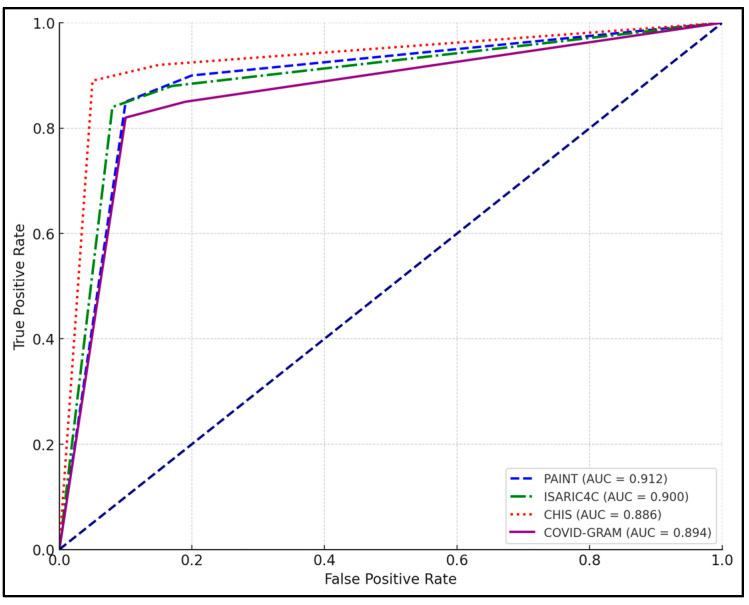
AUROC analysis of clinical prediction scores for COVID-19 mortality at one week post symptom onset.

One week post symptom onset, the predictive values of these scores increased, which was particularly evident in the elevated cutoff values and AUCs, suggesting that as the disease progresses, the scores become even more indicative of severe outcomes. The PAINT score’s cutoff rose to 8.15, with its sensitivity and AUC increasing to 90.12% and 0.912, respectively, indicating enhanced predictive accuracy over time. Similarly, the ISARIC4C score increased to a cutoff of 9.10 with a sensitivity of 87.98% and AUC of 0.900. Notably, the CHIS score showed the highest sensitivity at one week (91.67%), with an AUC of 0.886. The COVID-GRAM, designed to integrate clinical and laboratory parameters, also showed an improved performance with a cutoff of 0.72 and an AUC of 0.894 (Table 4 and Figure 2).

At baseline, the hazard ratios indicate the likelihood of severe COVID-19 development among those with scores above the best cutoff values. The PAINT score revealed a hazard ratio of 3.45 (95% CI: 2.10–5.67, *p* < 0.0001), suggesting that patients with diabetes who exceeded this score at baseline were over three times more likely to develop severe COVID-19 compared to those below the cutoff. The ISARIC4C score had a hazard ratio of 2.89 (95% CI: 1.85–4.50, *p* = 0.0003), and the CHIS score showed a more pronounced risk at 4.02 (95% CI: 2.56–6.30, *p* < 0.0001). The COVID-GRAM score also indicated significant risk at 3.15 (95% CI: 2.01–4.92, *p* = 0.0002), demonstrating its utility alongside the other scores in predicting severe outcomes at the point of patient admission.

One week after symptom onset, the hazard ratios increased, suggesting that the scores’ predictive capability strengthens as the clinical presentation evolves. The PAINT score rose to 4.88 (95% CI: 3.10–7.68, *p* < 0.0001), indicating that the risk of severe disease nearly quintuples for those scoring above this threshold compared to those below it. The ISARIC4C score’s hazard ratio increased to 3.67 (95% CI: 2.34–5.78, *p* < 0.0001), the CHIS score to 5.34 (95% CI: 3.45–8.21, *p* < 0.0001), and the COVID-GRAM to 4.22 (95% CI: 2.67–6.70, *p* < 0.0001), as presented in Table 5.

## 4. Discussion

### 4.1. Analysis of Findings

The critical findings of this study underscore the significant utility of the PAINT, ISARIC4C, CHIS, and COVID-GRAM scores in predicting severe outcomes in hospitalized COVID-19 patients, particularly those with comorbid diabetes. These tools demonstrated strong predictive accuracy at both admission and one week post symptom onset, with distinct cutoff values enhancing their application in clinical settings. Notably, the increased hazard ratios observed from baseline to one week suggest that the scores’ predictive power strengthens as the clinical condition of patients deteriorates, highlighting their importance in continuous patient monitoring.

At baseline, each of the scores identified patients at significantly increased risk of severe disease progression. For instance, the PAINT score, which integrates markers of pulmonary disease severity and immune response among other factors, showed a hazard ratio of 3.45, indicating that patients above this cutoff were more than three times as likely to experience severe outcomes compared to those below the threshold. Similarly, the ISARIC4C and CHIS scores, reflecting broader clinical and inflammatory markers, also indicated substantial risk elevations with hazard ratios of 2.89 and 4.02, respectively. This emphasizes their role not just in initial assessment but also in guiding early intervention strategies.

One week post symptom onset, the predictive capacity of these scores increased even further, aligning with the worsening clinical parameters observed in patients who eventually experienced severe outcomes. For example, the PAINT score’s hazard ratio rose to 4.88, suggesting a nearly fivefold increase in risk for severe disease among those surpassing this score threshold. This period is critical as it likely represents a pivotal point in disease progression where targeted therapeutic interventions could be most effective in altering patient trajectories towards recovery rather than deterioration.

In their respective studies, Wang et al. [17] and Ji et al. [21] both developed predictive scores for COVID-19 progression with high predictive accuracy. Wang et al. established the PAINT score, which includes factors such as pulmonary disease and age over 75, demonstrating a robust C-index of 0.91 (*p* < 0.001) in a cohort of 239 patients. This score effectively differentiated patients at risk of progressing from mild/moderate to severe COVID-19. Similarly, Ji et al. introduced the CALL score, utilizing variables like comorbidity and lymphocyte counts, achieving an area under the ROC of 0.91 and providing a well-fitted calibration curve across 208 patients. The CALL score, with a cutoff of six points, showed a positive predictive value of 50.7% and a negative predictive value of 98.5%, highlighting its utility in clinical decision-making.

In a similar manner, two studies conducted external validations of the ISARIC4C Mortality Score in different contexts, revealing varied performances in predicting in-hospital mortality among ICU patients with COVID-19. Vallipuram et al. [22] reported a good predictive performance of the ISARIC4C score in a Canadian ICU setting, with an area under the curve of 0.762, which outperformed the SOFA and APACHE II scores, which had AUCs of 0.705 and 0.722, respectively. This study, involving 429 patients with a mortality rate of 23.8%, demonstrated the score’s robustness in a severely ill population. Conversely, Durie et al. [23] in Australia found that the ISARIC4C score, although showing a comparable AUC of 0.79 to the original UK cohort’s 0.77, consistently overestimated the risk of death in their cohort of 461 patients, with an actual mortality of 13.2% against a predicted 22.9%. This discrepancy highlighted the potential need for recalibration of the ISARIC4C score when applied outside its initial development setting, particularly in different healthcare environments and population demographics.

In their studies on the applicability of the ISARIC-4C score for predicting severity and mortality in COVID-19 patients, Albai et al. [24] and De Vito et al. [25] explored the effectiveness of the score under varying conditions and patient demographics. Albai et al. focused on a cohort of 159 patients with type 2 diabetes, confirming the score’s robustness with an area under the curve of 0.875, demonstrating that patients with a score higher than 14 had significantly increased mortality risks (63.93% vs. 31.24%; *p* < 0.001). The study highlighted the ISARIC-4C score’s high sensitivity (87.80%) and specificity (73.72%) in this specific subgroup, making it a valuable tool for early prognostication and therapeutic strategy development. In a similar manner, De Vito et al. assessed the score’s validity during the Omicron variant wave among 1186 patients, showing a lower overall mortality rate (13.5%) but still a decent predictive capability with an AUC of 0.78. This study reinforced the ISARIC-4C score’s utility in identifying high-risk patients, despite variations in virus strains and clinical outcomes over time.

In their studies assessing the impact of hyperinflammation in COVID-19 patients, Hsu et al. [26] and Yildirim et al. [27] explored the association between the COVID-19-associated hyperinflammation score and clinical outcomes in specific patient populations. Hsu et al. conducted a retrospective cohort study focusing on patients with systemic rheumatic diseases, finding that such patients exhibited higher median values for inflammatory markers and faced increased risks for ICU admission and mechanical ventilation compared to matched controls without rheumatic diseases. Specifically, they observed that patients with a cHIS of 2 or more had significantly higher odds of severe outcomes including ICU admission (OR 3.45) and mechanical ventilation (OR 66.20), and in-hospital mortality (OR 16.37). Similarly, Yildirim et al. examined critically ill COVID-19 patients, finding that a cHIS score of ≥3 at admission was associated with worse outcomes, including higher ICU, hospital, and 28-day mortality rates. This study highlighted the predictive value of the cHIS score in forecasting severe clinical outcomes such as ICU mortality (sensitivity = 0.63, specificity = 0.50) and the requirement for invasive mechanical ventilation (sensitivity = 0.61, specificity = 0.51).

These findings are pivotal for refining clinical decision-making processes, especially in settings burdened by high caseloads and limited resources. By effectively stratifying patients based on their risk of severe complications, healthcare providers can prioritize care and allocate resources more efficiently, potentially lowering mortality rates. Moreover, the robust statistical significance of these results not only underscores the reliability of these prediction scores but also boosts their potential for widespread adoption in various healthcare systems. Thus, this study contributes valuable insights into the management of COVID-19, supporting the use of sophisticated predictive tools to improve patient outcomes during ongoing and future health crises.

### 4.2. Study Limitations and Future Perspectives

This study, while robust in its findings, is not without limitations. The retrospective nature of the analysis means that the results are inherently dependent on the accuracy and completeness of recorded data, which may limit the ability to control for all potential confounding variables. Additionally, the study was conducted in a single clinical setting, which may affect the generalizability of the results to other populations or healthcare systems with different patient demographics or treatment protocols. Future research should aim to validate these findings in prospective, multi-center studies that include a more diverse patient population. Moreover, integrating these prediction scores into real-time clinical decision support systems could provide dynamic risk assessments, thereby enhancing their practical utility in managing COVID-19 patients effectively.

## 5. Conclusions

The current study demonstrates the significant utility of the PAINT, ISARIC4C, CHIS, and COVID-GRAM scores in predicting severe outcomes in hospitalized COVID-19 patients, particularly those with diabetes. These prediction tools exhibited high sensitivity and specificity at baseline and one week post symptom onset, effectively identifying patients at increased risk for severe complications. The robust predictive power of these scores, as evidenced by significant hazard ratios and AUC values, supports their integration into clinical practice for timely and targeted intervention. Ultimately, these tools hold promise not only for managing COVID-19 but also for potential applications in future outbreaks of similar infectious diseases, underscoring the need for ongoing research and development in clinical predictive analytics.

## Figures and Tables

**Table 1:** Demographic and clinical characteristics of COVID-19 patients included in the study.

Variables	Survivors Group (*n* = 139)	Mortality Group (*n* = 76)	*p*-Value
Age, years (mean ± SD)	57.23 ± 14.56	60.89 ± 10.42	0.0542
Gender, men	78 (56.12%)	33 (43.42%)	0.1015
BMI (mean ± SD)	27.68 ± 4.89	30.12 ± 6.03	0.0010
Smoking	26 (18.71%)	22 (28.95%)	0.1205
Alcohol use	31 (22.30%)	27 (35.53%)	0.0539
COVID-19 vaccinated	89 (64.03%)	39 (51.32%)	0.0949
CCI > 2	42 (30.22%)	34 (44.74%)	0.0477
COVID-19 severity *	-	-	0.0954
Mild	95 (68.35%)	41 (53.95%)	
Moderate	29 (20.86%)	21 (27.63%)	
Severe	15 (10.79%)	14 (71.05%)	
ICU admissions	5 (3.60%)	41 (18.42%)	<0.0001
Supplemental oxygen	18 (12.95%)	64 (84.21%)	<0.0001
Mechanical ventilation	4 (2.88%)	39 (51.32%)	<0.0001
Mortality	0 (0%)	76 (100%)	<0.0001

*—Severity was assessed at admission; SD—Standard Deviation; BMI—Body Mass Index; CCI—Charlson Comorbidity Index; ICU—Intensive Care Unit.

**Table 2:** Physiological parameters and severity scores at admission.

Variables (Mean ± SD)	Survivors Group (*n* = 139)	Mortality Group (*n* = 76)	*p*-Value
Oxygen saturation (%)	94.32 ± 1.76	85.47 ± 3.29	<0.001
WBC (×10^9^/L)	6.47 ± 1.34	11.28 ± 4.26	<0.001
Lymphocyte count (×10^9^/L)	1.45 ± 0.48	0.82 ± 0.37	<0.001
IgM levels (mg/dL)	120.56 ± 35.12	69.89 ± 22.45	<0.001
CD16 (cells/µL)	352.67 ± 110.32	190.44 ± 89.15	<0.001
Respiratory rate (breaths/min)	18.47 ± 2.11	27.34 ± 5.78	<0.001
AST (U/L)	25.78 ± 8.96	58.44 ± 15.37	<0.001
Temperature (°C)	37.1 ± 0.46	38.5 ± 0.82	<0.001
Heart rate (bpm)	88.34 ± 12.34	103.47 ± 19.22	<0.001
Glasgow coma scale	14.78 ± 0.42	11.34 ± 2.86	<0.001
Bilirubin levels (mg/dL)	0.68 ± 0.22	1.45 ± 0.58	<0.001
IL-6 (pg/mL)	12.34 ± 4.67	46.87 ± 17.32	<0.001
D-dimers (mg/L FEU)	0.55 ± 0.25	3.98 ± 1.74	<0.001
Creatinine (mg/dL)	0.89 ± 0.18	1.34 ± 0.42	<0.001
Ferritin (ng/mL)	250.45 ± 110.78	742.89 ± 330.45	<0.001
CRP (mg/L)	20.78 ± 10.44	156.34 ± 70.56	<0.001
Platelets (×10^9^/L)	251.34 ± 50.12	120.89 ± 45.67	<0.001
Systolic blood pressure (mmHg)	130.67 ± 14.22	118.56 ± 20.45	<0.001
BUN (mg/dL)	15.34 ± 4.22	30.89 ± 11.34	<0.001
Clinical scores			
PAINT	3.25 ± 1.11	7.84 ± 2.56	<0.001
ISARIC4C	4.22 ± 1.34	11.45 ± 3.67	<0.001
CHIS	2.56 ± 1.22	8.34 ± 3.12	<0.001
COVID-GRAM	0.34 ± 0.12	0.89 ± 0.23	<0.001

SD—Standard Deviation; WBC—White Blood Cell; BUN—Blood Urea Nitrogen; PAINT—Pulmonary disease, Age, IgM, Natural killer (NK) cells (CD16+/CD56+), Transaminases (Aspartate Aminotransferase, AST); ISARIC4C—International Severe Acute Respiratory and emerging Infection Consortium Coronavirus Clinical Characterization Consortium Mortality Score; CHIS—COVID-19-associated Hyperinflammatory Syndrome; COVID-GRAM—COVID-GRAM Risk Score (where GRAM stands for the clinical and laboratory parameters included in the score, though GRAM itself is not an acronym).

**Table 3:** Clinical scores and physiological parameters at one week post symptom onset.

Variables (Mean ± SD)	Survivors Group (*n* = 139)	Mortality Group (*n* = 76)	*p*-Value
Oxygen saturation (%)	92.87 ± 2.15	84.35 ± 4.26	<0.001
WBC (×10^9^/L)	7.56 ± 2.03	15.27 ± 5.89	<0.001
Lymphocyte count (×10^9^/L)	1.65 ± 0.62	0.79 ± 0.30	<0.001
IgM levels (mg/dL)	158.78 ± 45.63	50.12 ± 28.57	<0.001
CD16 (cells/µL)	420.58 ± 135.77	163.39 ± 102.54	<0.001
Respiratory rate (breaths/min)	17.34 ± 3.12	28.67 ± 7.54	<0.001
AST (U/L)	23.45 ± 9.87	70.98 ± 25.34	<0.001
Temperature (°C)	37.2 ± 0.55	38.9 ± 1.12	<0.001
Heart rate (bpm)	86.23 ± 13.45	110.56 ± 23.45	<0.001
Glasgow coma scale	14.89 ± 0.87	9.78 ± 3.56	<0.001
Bilirubin levels (mg/dL)	0.78 ± 0.34	2.65 ± 1.29	<0.001
IL-6 (pg/mL)	15.67 ± 6.45	80.23 ± 35.67	<0.001
D-dimers (mg/L FEU)	0.75 ± 0.38	7.45 ± 3.89	<0.001
Creatinine (mg/dL)	0.97 ± 0.26	2.35 ± 1.08	<0.001
Ferritin (ng/mL)	320.34 ± 150.78	1520.89 ± 620.45	<0.001
CRP (mg/L)	30.56 ± 15.47	250.78 ± 120.56	<0.001
Platelets (×10^9^/L)	230.45 ± 75.12	85.67 ± 50.34	<0.001
Systolic blood pressure (mmHg)	128.34 ± 18.22	95.67 ± 25.45	<0.001
BUN (mg/dL)	18.34 ± 5.67	45.12 ± 20.89	<0.001
Clinical scores			
PAINT	2.87 ± 1.22	9.34 ± 3.45	<0.001
ISARIC4C	3.65 ± 2.11	14.56 ± 4.98	<0.001
CHIS	1.98 ± 1.08	12.67 ± 5.34	<0.001
COVID-GRAM	0.45 ± 0.23	0.98 ± 0.32	<0.001

SD—Standard Deviation; WBC—White Blood Cell; BUN—Blood Urea Nitrogen; PAINT—Pulmonary disease, Age, IgM, Natural killer (NK) cells (CD16+/CD56+), Transaminases (Aspartate Aminotransferase, AST); ISARIC4C—International Severe Acute Respiratory and emerging Infection Consortium Coronavirus Clinical Characterization Consortium Mortality Score; CHIS—COVID-19-associated Hyperinflammatory Syndrome; COVID-GRAM—COVID-GRAM Risk Score (where GRAM stands for the clinical and laboratory parameters included in the score, though GRAM itself is not an acronym).

**Table 4:** Best cutoff values for severe COVID-19 prediction in patients with diabetes.

Parameters	Timeframe	Best Cutoff Value	Sensitivity	Specificity	AUC	*p*-Value
PAINT	Baseline	6.26	85.47	77.34	0.861	<0.0001
ISARIC4C	Baseline	7.95	80.56	82.12	0.879	<0.0001
CHIS	Baseline	5.58	88.89	75.01	0.842	<0.0001
COVID-GRAM	Baseline	0.63	83.33	78.45	0.851	<0.0001
PAINT	One week	8.15	90.12	79.56	0.912	<0.0001
ISARIC4C	One week	9.10	87.98	81.67	0.900	<0.0001
CHIS	One week	7.84	91.67	74.56	0.886	<0.0001
COVID-GRAM	One week	0.72	85.45	80.34	0.894	<0.0001

PAINT—Pulmonary disease, Age, IgM, Natural killer (NK) cells (CD16+/CD56+), Transaminases (Aspartate Aminotransferase, AST); ISARIC4C—International Severe Acute Respiratory and emerging Infection Consortium Coronavirus Clinical Characterization Consortium Mortality Score; CHIS—COVID-19-associated Hyperinflammatory Syndrome; COVID-GRAM—COVID-GRAM Risk Score (where GRAM stands for the clinical and laboratory parameters included in the score, though GRAM itself is not an acronym).

**Table 5:** Regression analysis for severe COVID-19 development in patients with diabetes.

Factors above the Best Cutoff	Timeframe	Hazard Ratio	95% CI	R^2^	*p*-Value
PAINT	Baseline	3.45	2.10–5.67	0.74	<0.0001
ISARIC4C	Baseline	2.89	1.85–4.50	0.69	0.0003
CHIS	Baseline	4.02	2.56–6.30	0.71	<0.0001
COVID-GRAM	Baseline	3.15	2.01–4.92	0.66	0.0002
PAINT	One week	4.88	3.10–7.68	0.76	<0.0001
ISARIC4C	One week	3.67	2.34–5.78	0.73	<0.0001
CHIS	One week	5.34	3.45–8.21	0.81	<0.0001
COVID-GRAM	One week	4.22	2.67–6.70	0.79	<0.0001

PAINT—Pulmonary disease, Age, IgM, Natural killer (NK) cells (CD16+/CD56+), Transaminases (Aspartate Aminotransferase, AST); ISARIC4C—International Severe Acute Respiratory and emerging Infection Consortium Coronavirus Clinical characterization Consortium Mortality Score; CHIS—COVID-19-associated Hyperinflammatory Syndrome.

## Data Availability

The data presented in this study are available on request from the corresponding author (the data are not publicly available due to privacy or ethical restrictions).
